# Discovery of Functional SNPs via Genome-Wide Exploration of Malaysian Pigmented Rice Varieties

**DOI:** 10.1155/2019/4168045

**Published:** 2019-10-10

**Authors:** Rabiatul-Adawiah Zainal-Abidin, Norliza Abu-Bakar, Yun-Shin Sew, Sanimah Simoh, Zeti-Azura Mohamed-Hussein

**Affiliations:** ^1^Centre for Bioinformatics Research, Institute of Systems Biology (INBIOSIS), Universiti Kebangsaan Malaysia (UKM), 43600 UKM Bangi, Selangor, Malaysia; ^2^Malaysian Agricultural Research & Development Institute (MARDI), Persiaran MARDI-UPM, 43300 Serdang, Selangor, Malaysia; ^3^Centre for Frontier Sciences, Faculty of Science & Technology (FST), Universiti Kebangsaan Malaysia (UKM), 43600 UKM Bangi, Selangor, Malaysia

## Abstract

Recently, rice breeding program has shown increased interests on the pigmented rice varieties due to their benefits to human health. However, the genetic variation of pigmented rice varieties is still scarce and remains unexplored. Hence, we performed genome-wide SNP analysis from the genome resequencing of four Malaysian pigmented rice varieties, representing two black and two red rice varieties. The genome of four pigmented varieties was mapped against Nipponbare reference genome sequences, and 1.9 million SNPs were discovered. Of these, 622 SNPs with polymorphic sites were identified in 258 protein-coding genes related to metabolism, stress response, and transporter. Comparative analysis of 622 SNPs with polymorphic sites against six rice SNP datasets from the Ensembl Plants variation database was performed, and 70 SNPs were identified as novel SNPs. Analysis of SNPs in the flavonoid biosynthetic genes revealed 40 nonsynonymous SNPs, which has potential as molecular markers for rice seed colour identification. The highlighted SNPs in this study show effort in producing valuable genomic resources for application in the rice breeding program, towards the genetic improvement of new and improved pigmented rice varieties.

## 1. Introduction

Rice (*Oryza sativa* L.) is the most crucial staple food crops in Asian countries. The most consumed rice is white rice, which resulted from the white pericarp. The coloured pericarp such as black, red, and brown has become more popular. Coloured pericarp accumulates secondary metabolites such as flavonoid, anthocyanin, and proanthocyanidin and usually are associated as potent antioxidants. Previous study has found that food sources with high antioxidant properties can lower the risk of chronic diseases such as type II diabetes, cardiovascular disease, and cancers [1]. Hence, this finding has accelerated the development of pigmented rice varieties.

Previous efforts have been performed to elucidate the genetic basis of black and red rice varieties [2–4]. In red rice variety, *Rc* is responsible for the accumulation of proanthocyanidins in red pericarp, but it has to interact with *Rd* gene that encodes for dihydroflavonol-4-reductase (DFR) that involved in the catalysis activity of dihydroflavonol to leucoanthocyanidin [2, 3]. However, without this interaction, brown rice will be produced whilst *Rd* alone has no phenotype change. *Rc* is also known as domestication gene [5] and has been widely used to investigate the domestication process in rice subspecies [6–8]. *Kala4*, a transcription factor in basic helix-loop-helix (bHLH) family, is involved in black rice pigmentation [4]. Ectopic expression in *Kala4* causes the upregulation of LDOX in pericarp, accumulates the anthocyanidin, and produces black pericarp [4].

To further investigate the genetic basis of pigmented rice varieties, many efforts have been performed using omics technologies and bioinformatics. For instance, several studies on the phytochemical diversity of the coloured or pigmented rice from landraces, varieties, and wild relatives have been widely conducted using a metabolomics approach to reveal their antioxidant properties and variabilities [9–14]. Previous studies on the transcriptome sequencing of pigmented rice varieties were conducted to identify single-nucleotide polymorphisms (SNPs) and regulatory genes, which might be responsible in the accumulation of anthocyanin [15, 16]. An integrative omics approach, combining proteomics and transcriptomics sequencing, was conducted to identify the flavonoid biosynthetic genes in the black and red rice varieties [17] and potential biomarkers responsible to the accumulation of flavonoid in rice varieties by linking the SNP located in the flavonoid biosynthetic genes to flavonoid accumulation [18]. Meanwhile, genome resequencing of pigment rice varieties has been performed to identify potential SNPs located in the biosynthetic genes, which can be developed as molecular markers for nutritional quality traits such as high antioxidant [19, 20] and high amylose content [21]. All these efforts showed the importance of mining genetic variant, biosynthetic genes, and transcription factors in order to understand the interactions that will affect and influence the biosynthesis of antioxidant contents in rice varieties.

Molecular marker is a DNA fragment with phenotypic expression that is associated with a certain location within the genome [22]. Several types of molecular markers such as random amplified polymorphic DNA (RAPD), restriction fragment length polymorphism (RFLP), and microsatellite (SSR) are widely used in the genetic improvement of rice [23]. Recently, the application of SNP in rice breeding improvement is rapidly expanding. The combinatorial approach between the next-generation sequencing technology (NGS) and bioinformatics has greatly assisted SNPs' discovery from the genome, followed by the validation of SNPs conducted using current genotyping technology [24]. Thus, the application of bioinformatics in predicting SNPs from the genome sequences is crucial to accelerate the implementation of genome-based breeding approaches for the development of rice varieties with desirable agronomical traits [25].

SNP is defined as a single base difference in DNA sequence and the most common type of genetic variation to distinguish individuals [26]. The abundance of SNPs in the genome can be used in the improvement of high-resolution genetic map that will lead to the association of SNP with agronomic traits of interest [27]. Interestingly, SNPs located in the genic region could affect the phenotypic expression of crops and are applicable for gene functional analysis and marker-assisted selection (MAS) [28]. SNPs have been applied to investigate the evolution and domestication of rice [29–31] and the identification of functional SNP in genes related to various agronomic traits such as domestication trait [32], seed size [33], salinity tolerance [34] and response to stress [35], diversity analysis among cultivars [36–39], and seed purity assessments [40]. These efforts showed the utilisation of SNP for rice breeding improvement. However, not much effort has been conducted to explore the genetic variation in Malaysian pigmented rice varieties using single-nucleotide polymorphism (SNP). As a result, this has to limit genetic understanding of pigmented rice that is crucial for the genetic improvement of pigmented rice varieties.

**Figure 1:**
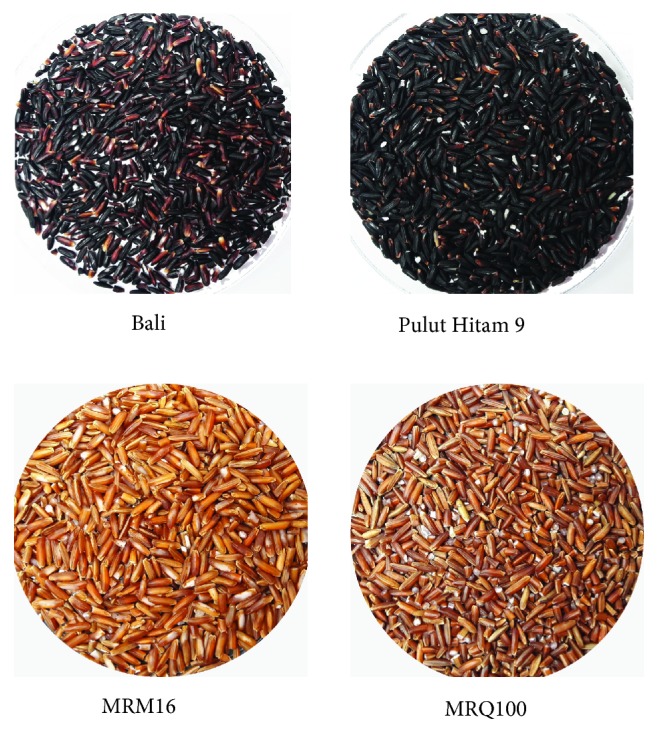
Whole grains of Bali, Pulut Hitam 9, MRM16, and MRQ76. Pulut Hitam 9 has a darker black pigment compared to Bali, while MRM16 has a darker red pigment compared to MRQ100.

Here, we report the genome-wide SNP analysis on the whole genome resequencing of two black rice varieties (Bali and Pulut Hitam 9) and two red rice varieties (MRM16 and MRQ100). Bali is a landrace rice variety, while Pulut Hitam 9 (PH9), MRM16, and MRQ100 are modern rice varieties. All of them were from indica subspecies. These four varieties were chosen due to their nutritional trait that was enriched with antioxidant properties [14].  Figure 1 shows the whole grains of Bali, Pulut Hitam 9, MRM16, and MRQ100.

We mined the SNPs from the genomes of four pigmented Malaysian rice varieties to search for the SNPs with polymorphic sites and candidate SNPs associated with the flavonoid biosynthetic genes. Additionally, we have identified 70 novel SNPs after comparing with SNP data from Ensembl Plants variation [41], comprising the variation data from six large-scale SNP studies. The SNPs highlighted in this study are suggested as potential molecular markers for further validation using a genotyping platform, towards genetic improvement of pigmented rice varieties.

## 2. Materials and Methods

### 2.1. Plant Materials

Plant materials consisted of four pigmented rice varieties from Malaysian, i.e., Bali, PH9, MRM16, and MRQ100. Four varieties were selected based on (a) the presence of high antioxidant contents and (b) released variety. Seeds of Bali, PH9, MRM16, and MRQ100 were obtained from MARDI Seberang Perai, Penang, Malaysia. Seeds were sterilized, incubated at 42°C overnight, and soaked in water for two days before being placed onto wet tissues or directly sowed into the soil.

### 2.2. DNA Isolation and Genome Sequencing

Total DNA of each variety was extracted from leaves of two-week-old germinated seedling using Mutou et al.'s protocol [42] and Sigma DNA extraction kit. DNA quality and quantity were analysed using NanoDrop spectrophotometer. The integrity of DNA samples was determined using 0.8% agarose gel. The DNA samples were sequenced using Illumina HiSeq 4000 sequencing (Illumina, Inc., San Diego, CA, USA). Standard Illumina protocol was used for the sequencing process.

### 2.3. Reads Mapping and Identification of SNPs

The pair-end sequencing reads from Bali, PH9, MRM16, and MRQ100 with the read length of 150 bp at each end were aligned with Nipponbare genome sequences [43] using Burrows-Wheeler Aligner (BWA) [44] software using default parameters except for “mem -m 10000 -o 1 -e 10 -t 4”. All genomes were individually aligned. The mapped reads were merged and indexed as BAM files. The mapped reads from each variety were then processed for mark duplicate reads, fixing mate-pair information, and adding or replacing read groups using PICARD version 0.7.12.

We followed the GATK best-practices pipeline for SNP calling [45]. This SNP-calling pipeline has been used in rice SNP discovery [31, 34, 46, 47] and development of SNP panel using genotyping platforms [48–50]. Local realigment and base quality score recalibration were performed on processed mapped reads using GATK version 3.6 [45]. By following these steps, false-positive SNPs can be reduced and it can increase the possibility to obtain reliable SNPs [51, 52]. SNP calling for each variety was independently conducted using the HaplotypeCaller package in (GATK) version 3.6 with a minimum phred-scaled confidence threshold of 50 and a minimum phred-scaled confidence threshold for emitting variants at 10. To ensure the quality of the SNP calling, the conditions for every site in a genome were set at (a) >30 for mapping quality, (b) >50 for variant quality, and (c) >10 for the number of supporting reads for every base. Another two criteria also were performed after SNPs calling, i.e., (i) distance between SNP and another SNP is >150 bp and (ii) SNP with a PASS score.

### 2.4. Annotation and Functional Classification of SNPs

SnpEff [53] version 4.1 was used to annotate SNPs into intergenic and genic. The genic SNPs were classified as coding sequences (CDS), untranslated region (UTR), and intron. SNPs in the CDS region were further divided into synonymous and nonsynonymous amino acid substitutions. Annotated SNPs were filtered accordingly with reference to the above criteria using R packages (dplyr, sqldf, and tidyr). Genomic distribution of SNPs was performed using R scripts and visualised using Flapjack [54]. Unique SNP in each variety was extracted using R scripts. The number of SNPs in CDS was counted using R scripts.

### 2.5. Enrichment Analysis

Gene ontology enrichment analysis of genes containing 622 SNPs with polymorphic sites was performed using PANTHER (protein annotation through evolutionary relationship) classification system [55] (http://www.pantherdb.org) with FDR cutoff selected at ≤0.05. Gene Ontology database for *Oryza sativa* was selected for this analysis.

### 2.6. Identification of SNP Genes Involved in the Flavonoid Biosynthetic Genes (FBGs)

The flavonoid biosynthetic genes (FBGs) were obtained from the similarity and bibliomic search. The list of FBGs is provided in the Supplementary [Supplementary-material supplementary-material-1]. Genic SNPs from each variety were compared to the flavonoid biosynthetic genes by matching with the *Oryza sativa* gene identification (OsID) using R scripts.

## 3. Results and Discussion

### 3.1. Mapping of Bali, PH9, MRM16, and MRQ100 Genome Data onto the Nipponbare Reference Genome

Genome sequencing of Bali, PH9, MRM16, and MRQ100 has produced 101.71, 99.98, 98.76, and 99.99 million reads, respectively. The average read lengths of 2 × 150 bp were generated with 30× depth of sequencing. This 30× depth of sequencing was chosen as it provides sufficient coverage in identifying high-quality genetic variations such as SNP, single-nucleotide variation (SNV), and insertion-deletion (InDel) [56]. Therefore, the relationship between the depth of sequencing and identification of SNPs is a key factor in obtaining high-quality SNPs. A total of 96.47% of Bali, 95.97% of PH9, 98.07% of MRM16, and 94.42% of MRQ100 million clean reads was obtained after the sequence read cleaning process. The clean reads for each variety were then mapped against the Nipponbare reference genome. Nipponbare was used as a reference genome sequence because it is well-assembled and annotated genome [34, 35, 57]. The mapped reads against Nipponbare genome showed that almost 96% of the reads were successfully mapped onto the rice genome. Low divergence of genetic differences between i*ndica* and *japonica* varieties might be a contributing factor that caused the highest mapped rate.  Table 1 represents a summary of the sequence reads and mapping data in four pigmented rice varieties.

### 3.2. Identification of SNPs and SNPs with Polymorphic Sites

Table 2 provides statistics of raw and high-quality SNPs for Bali, PH9, MRM16, and MRQ100 genome. MRM16 contained the highest variation among the genomes, suggesting that MRM16 has a distant relationship to Nipponbare.

**Figure 2:**
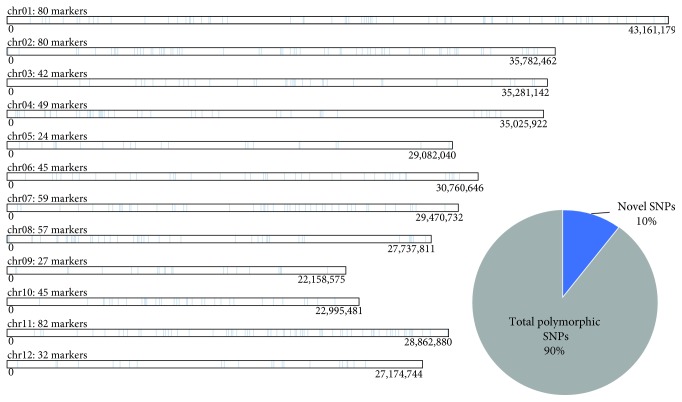
Distribution of 662 SNPs with polymorphic sites on 12 rice chromosomes. Of these, 70 novel SNPs (10%) were detected when compared against *Oryza sativa* japonica Ensembl Plants variation database.

Figure 2 shows the distribution of 662 SNPs with polymorphic sites on 12 rice chromosomes. SNPs with polymorphic sites are defined as the presence of SNP in the individual but with several different alleles. A set of SNPs with polymorphic sites indicates that the SNP is highly informative, thus suitable as a potential candidate for genetic marker development [58]. Supplementary [Supplementary-material supplementary-material-1] shows the character of SNPs with polymorphic sites.

Distribution of these polymorphic sites on the 12 rice chromosomes shows that chromosome 11 consisted of the highest number of SNPs with polymorphic sites (82), followed by chromosome 1 (80) and chromosome 2 (80). These values demonstrate the random distribution of SNPs with polymorphic sites within the 12 rice chromosomes. Interestingly, 70/10% of the SNPs with polymorphic sites were novel SNPs based on the comparison against *Oryza sativa* Ensembl Plants variation database as of October 2017 (Figure 1). The SNP datasets in the *Oryza sativa* Ensembl Plants variation were from six large-scale SNP studies [59–64]. This finding indicates that many SNPs have been discovered from various rice cultivars by rice genome-sequencing effort from time to time. The 70 novel SNPs with polymorphic sites can be suggested as molecular markers for varietal identification.

### 3.3. Annotation of SNPs and SNPs with Polymorphic Sites

The annotation of SNPs in four pigmented rice varieties has revealed that most of the SNPs were located in the intergenic region (1,315,780; 64%) while fewer SNPs are located within the genic region (604,936; 29%) (Table 2). This finding corroborated with the results obtained by Tatarinova et al. where the SNP rate is higher in the intergenic regions compared to that in the genic regions [65]. This finding is common in SNP discovery as the coding regions are more conserved than intergenic regions [65].

Analysis of the SNP differences between rice varieties showed that MRM16 (165,124) has a higher number of SNPs in the genic region whereas PH9 (140,677) has the least number of SNPs in the genic region. High number of SNPs in the genic region of MRM16 suggested the introgression, and recombination have occurred through human-guided artificial selection during rice breeding activity. Previous studies by Sang et al. and Tatarinova et al. suggested that artificial selection in developing modern rice varieties has shaped the present of SNP frequency and gene pool in the rice genome [5, 65].

Functional annotation analysis was performed to explore the effect of 662 SNPs with polymorphic sites on gene function. SNPs with polymorphic sites in the genic region will be valuable if associated with phenotypic expression or important agronomical trait [28]. Enrichment analysis based on the Gene Ontology (GO) terms was conducted on the 662 SNPs with polymorphic sites for functional annotation towards investigating their effect on the gene function. The top ten GO terms from biological processes and molecular function terms have been chosen for further discussion (Table 3).

GO:0009987 (cellular process) and GO:0008152 (metabolic process) were assigned for all genes that carry the SNPs with polymorphic sites in Bali, PH9, MRM16, and MRQ100 varieties suggesting their involvement in various physiological functions. Cellular process plays essential roles in cell communication while the metabolic process involved in the anabolism and catabolism of biosynthesis pathway. In the molecular function category, the SNPs with polymorphic sites were assigned to the binding function (heterocyclic compound binding, organic cyclic compound binding, ion binding, small molecule binding, and carbohydrate derivative binding) and catalytic activity suggesting their possible involvement in the formation of molecule and enzymatic activities related to abiotic stress [34], several biochemical pathways, and disease trait [66].

The biological interpretation of genes in the SNPs with polymorphic sites was further examined using the information obtained from the Reactome pathway analysis [67]. In total, three major pathways were found to be correlated with the top 10 GO terms, such as metabolism and regulation (R-OSA-2744345), secondary metabolite biosynthesis (R-OSA-2744341), and hormone biosynthesis, signalling, and transport (R-OSA-2744341) (Table 3). This finding corroborates with a study by Lin et al. that most of the SNPs and genes in the pigmented rice varieties were abundant in metabolic pathways such as flavonoid and anthocyanin biosynthetic pathways [19]. Hence, SNPs with polymorphic sites and genes in the pigmented rice genome might play an important role in the production of anthocyanin and proanthocyanidin. Our finding confirms the existence of phenotypic characteristic in pigmented rice (Bali, PH9, MRM16, and MRQ100) that are highly abundant with their antioxidant properties [14].

Functional annotation of the SNPs with polymorphic sites was further conducted using Pfam analysis on the 23 nonsynonymous SNPs (nsSNPs). Usually, nonsynonymous SNPs can affect the function of a gene to encode for the right protein, hence will affect its function. 13 nsSNPs were assigned into several functional gene classifications such as metabolism, stress response, and transporter and 10 nonsynonymous SNPs were assigned to the domain of unknown function (DUF).  Table 4 shows the annotation of 13 nsSNPs into their gene classifications.

Parida et al. discovered the involvement of Os01g0128000, Os07g0117000, Os09g0314200, Os10g0371100, and Os11g0539000 genes in plant resistance, pathogenesis, and abiotic stress mechanism [66]. Our analysis has identified that all the above genes have one nsSNP while two nsSNPs were found in Os01g0147001 that encodes for glycosyltransferase family 43 enzymes (important in the biosynthesis of cell wall [68] and Os02g0503900 that encodes for a cytochrome P450 (involved in xylan biosynthesis [69], two nsSNPs were also found in Os06g0695800 that encoded for ATP-binding cassette (ABC) transporter genes (important in iron intake for the improvement of plant micronutrient content [70] and were involved in the transportation of molecules, secondary metabolites, and plant hormones [71]). Further investigation on these genes is recommended to reveal the specific role of these variants in plant development and defence system.

Besides, four nsSNPs were also identified in four transcription factor families such as Myb-like DNA-binding domain (Os01g0128000), AP2 domain (Os10g0371100), IQ calmodulin-binding motif (Os07g0562800), and SWItch/Sucrose Non-Fermentable (SWI/SNF2) family N-terminal domain (Os08g0180300). Interestingly, Os01g0128000 that encodes for the Myb-like DNA-binding domain has been identified to be involved in the uptake and higher accumulation of phosphate (Pi) [72]. In particular, this gene was observed as a regulator in the cross-talk between nutrient signalling and phytohormone signalling pathway. Li et al. has reported that Os08g0180300 encodes for SWI/SNF2 and it is able to suppress rice innate immunity thus remarkably important in the defence mechanism against pathogen attack [73]. Hence, variation in these genes might affect the disease resistance capability of rice.

On the contrary, not much study has been conducted to confirm the function of Os10g0371100 that encodes for the ethylene-responsive transcription factor (ERF) domain or AP2/ERF domain. However, Os10g0371100 is predicted to be involved in plant growth and development either as an activator or a repressor in the expression of stress-responsive genes that are related to the abiotic stress responses [74]. Similarly, not much work has been conducted on the function of Os07g0562800 that encodes for the IQ calmodulin-binding motif in rice. Nevertheless, this gene was predicted to play a role in regulating plant responses in the signal transduction pathway during biotic or abiotic stress condition [75]. Analysis of SNPs with polymorphic sites can facilitate the identification of candidate SNPs and genes for functional markers in traits related to nutritional, nutraceutical and disease that can be used in the marker-assisted selection (MAS) of pigmented rice varieties.

### 3.4. Identification of SNPs Associated with Flavonoid Biosynthetic Genes (FBGs)

Pigmented rice is significantly associated with higher antioxidant content due to the presence of anthocyanin and proanthocyanidin. The production of these secondary metabolites is controlled by a set of flavonoid biosynthetic genes such as DFR, LAR, ANR, UGT, and LDOX, which lead to the production of anthocyanin and proanthocyanidin. The difference between anthocyanin and proanthocyanidin synthesis is the inclusion of the catalysed enzymes LAR and ANR for proanthocyanidin, while catalysis of LDOX for anthocyanin. Besides, *Kala4* gene activates LBG to produce anthocyanin whilst *Rc* gene activates DFR to produce proanthocyanidin. *Rc* is unable to regulate the production of proanthocyanidin alone; instead, it requires the presence of *Rd* gene which encodes DFR to activate the accumulation of proanthocyanidin.

In this study, a total of 99 flavonoid biosynthetic genes (FBGs) were selected from Nipponbare genome using similarity and bibliomic search [76–81]. Supplementary [Supplementary-material supplementary-material-1] shows the list of 99 FBGs into three groups, i.e., (i) general phenylpropanoid (phenyalanine ammonia-lyase (PAL); cinnamic acid 4-hydroxylase (C4H); 4-coumarate CoA ligase (4CL)); (ii) early biosynthetic genes (EBG) (chalcone synthase (CHS); chalcone isomerase (CHI); flavanone 3-hydroxylase (F3H); flavanone 3′-hydroxylase, F3′H); and (iii) late biosynthetic genes (LBG) (dihydroflavonol reductase (DFR); leucoanthocyanidin reductase (LAR); UDP-glucose flavonoid 3-O-glucosyl transferase (UGT); leucoanthocyanidin oxidase (LDOX)) [82, 83]. Three transcription factors involved in the production of anthocyanin and proanthocyanidin were selected, i.e., R2R3-MYB, *Kala4*, and *Rc*. R2R3-MYB (Os06g0205100) due to their role in activating the DFR gene in the upstream biosynthesis [84, 85]. *Kala4* (Os04g0557500) encodes for a basic helix-loop-helix (bHLH) transcription factor, which plays a role in activating the LDOX gene in the regulation of black pigmentation [4]. *Rc* (Os07g0211500) has previously been shown as an activator for *Rd* (Os01g0633500) in the production of red pigmentation [2, 3].

A total of 1649 genic SNPs were found in the flavonoid biosynthetic genes, and 511 SNPs were identified in the genes related to the general phenylpropanoid, 463 SNPs in EBGs and 675 SNPs in LBGs (Table 5). A high number of variations was found in LBG due to a difference in patterns of evolutionary rate. A previous study has revealed that the upstream genes have been observed to evolve slower than downstream genes in the secondary metabolite biosynthesis [86]. A similar pattern has been observed in mango with a high number of variations in the downstream genes of the flavonoid biosynthetic pathway [87]. This finding suggests that mutations in the flavonoid biosynthetic genes could affect the accumulation of secondary metabolite end products such as anthocyanin and proanthocyanidin.

Interestingly, ten genic SNPs associated with UGT (Os02g0589400) were identified in this analysis. A previous study has reported that one SNP was strongly associated with UGT (Os02g0589400) and was suggested as a metabolite quantitative trait loci (mQTL) for antioxidant trait [88]. UDP-glucose flavonoid 3-O-glucosyl transferase (UGT) is an enzyme involved in the glycosylation process and is essential for pigment stabilisation and secondary metabolites storage [77]. For this reason, the variation in UGT might provide the possibility of finding the candidates for functional markers in the accumulation of antioxidant. However, further investigation is required to determine the actual function of these SNPs.

Two genic SNPs associated with UGT (Os01g0736300) at position 30712175 (chr01_30712175) and 30713739 (chr01_30713739) have been identified and were found as SNPs in the untranslated (UTR) region and CDS, respectively. This finding suggests that the mutation in the UGT can be used as potential genetic markers for the accumulation of antioxidant properties in the pigmented rice varieties as Dong et al. found that a mutation in Os01g0736300 was associated with 7-0-glycosylated flavonoids [18]. Furthermore, SNP (chr01_30713739) was predicted as a nonsynonymous SNP that is involved in amino acid substitution and might affect the protein function that leads to the phenotypic consequences.

In addition, there were 160 genic SNPs found in the transcription factor genes, i.e., 30 mutations in *Rc* (Os07g0211500), 38 mutations in R2R3-MYB genes, and 92 mutations in *Kala4* (Os04g0557500). In comparison to the number of SNPs in the structural genes, fewer SNPs were found in the transcription factor, and this finding suggests that the character of the transcription factors are highly conserved compared to other classes of genes [89]. In conclusion, polymorphism in the transcription factor plays a crucial role in the biosynthetic pathway as it is responsible for regulating the functions of biosynthetic genes and affecting the production of secondary metabolites [86, 87].

### 3.5. Comparative Analysis on Genic SNPs in Flavonoid Biosynthetic Genes among Bali, PH9, MRM16, and MRQ100

**Figure 3:**
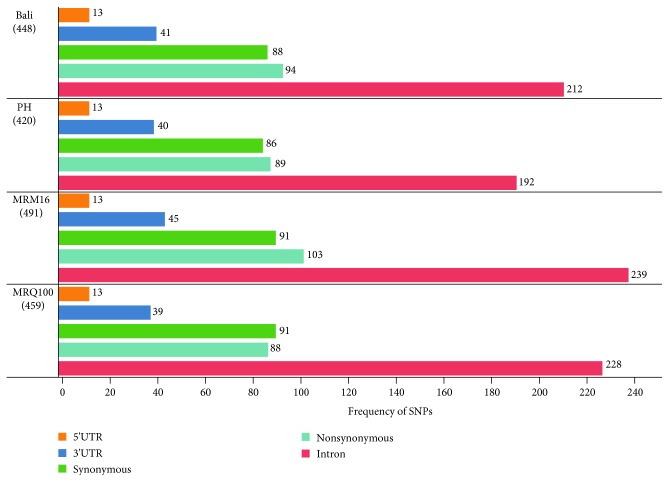
Distribution of genic SNPs identified in the flavonoid biosynthesis-related genes of Bali, PH9, MRM16, and MRQ100.

This study also investigated the distribution of genic SNPs in four pigmented rice varieties. A total of 448, 420, 491, and 459 genic SNPs were identified in Bali, PH9, MRM16, and MRQ100, respectively (Figure 3). Of these, 94, 89, 103, and 88 nonsynonymous SNPs (nsSNPs) were identified from Bali, PH9, MRM16, and MRQ100, respectively (Figure 3).

**Figure 4:**
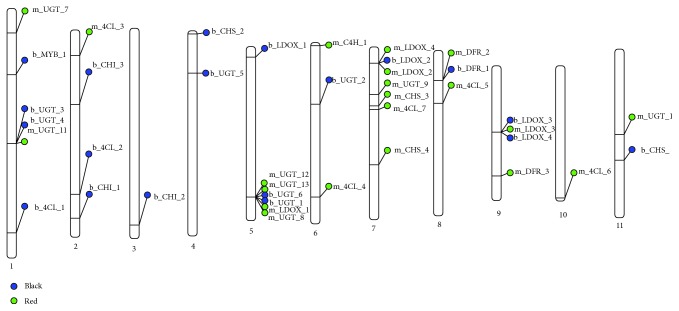
Physical positions of 40 nonsynonymous SNPs (nsSNPs) in the 39 flavonoid biosynthetic genes (FBGs) and one transcription factor. Blue circles represent black rice whereas green circles represent red rice. All nsSNPs were distributed on chromosome 1 to chromosome 11. None of the nonsynonymous SNPs reported in chromosome 12. SNP identifier (SNP ID) are listed on the right side of the blue and green circles.

SNPs are considered unique if they are present in one variety but absent in the other three varieties (Supplementary [Supplementary-material supplementary-material-1]). Hence, unique SNPs can be used to investigate the relationship between accessions and varieties [50]. In this study, a total of 40 nsSNPs in 39 flavonoid biosynthetic genes and one transcription factor was found unique to all four accessions (Figure 4 and Supplementary [Supplementary-material supplementary-material-1]). Supplementary [Supplementary-material supplementary-material-1] provides list of 40 nsSNPs and their SNPs information (i.e., SNP identifier (SNP ID), gene identifier, reference allele, SNP allele, chromosome, and SNP position).

The proportion of unique nsSNPs in these four varieties is lower, which is 10%. This finding suggests that these four varieties might share a common ancestor and may share similar genetic characteristics. The impact of unique variants has been demonstrated in wild strawberry where the occurrence of the genetic changes has caused the yellow colour phenotypic differences in three strawberry accessions [50].

Four unique nsSNPs (m_UGT_12, m_UGT_13, b_UGT_6, and b_UGT_1) were identified at positions 26199225, 26199416, 26199448, and 26199529 in UGT (Os05g0527000), respectively, and one nsSNP (b_UGT_2) which occurred at position 10479849 in UGT (Os06g0288300) (Figure 4). Os05g0527000 and Os06g0288300 that encoded for UGT have been reported as potential markers to distinguish different accumulations of flavonoid in *Indica* subspecies [88]. Finally, one nonoverlap nsSNP has been found in Os01g0305900 that encodes for R2R3-MYB (b_MYB_1), which is a transcription factor, and this unique nsSNP can only be found in the black rice variety Pulut Hitam 9. This unique nsSNP can be used as a potential genetic marker for rice seed colour identification.

Genomic variation among these four pigmented rice varieties provides a resource for genetic variability as well as generating new allelic variants towards the development of new and improved pigmented rice varieties. However, SNP validation must be conducted using a genotyping platform. This genome-wide gene-based SNP marker identification can provide a solution for breeders to effectively screen diverse accessions or interspecific hybrid breeding program for the genetic improvement in pigmented rice varieties.

## 4. Conclusions

Extensive bioinformatic analysis on next-generation sequencing (NGS) data has contributed to the identification of a high number of SNPs. From this study, the candidate SNPs associated with the essential functional genes and SNPs with polymorphic sites provide important insights into the genetic basis of four Malaysian pigmented rice varieties. Therefore, a genotyping experiment can be conducted on these SNPs for validation before progressing into genetic diversity study, cultivar identification, and marker-assisted selection (MAS), towards the development of new and improved pigmented rice varieties.

## Figures and Tables

**Table 1:** Summary of sequence reads and mapping statistics in Bali, PH9, MRM16, and MRQ100 genome.

	Bali	PH9	MRM16	MRQ100
Total reads (bp)	101,710,572	99,980,328	98,764,058	99,998,624
Number of clean reads (bp)	99,865,228 (98.18%)	99,380,446 (99.40%)	98,078,122 (99.30%)	94,428,632 (99.43%)
Genome coverage (30×)	88.59%	88.45%	88.45%	88.49%
Total mapped reads	96,479,796	95,971,696	94,870,967	91,170,844
Percentage of total mapped reads	96.61%	96.57%	96.73%	96.55%

**Table 2:** Summary of SNP identification and annotation in Bali, PH9, MRM16, and MRQ100 when compared against Nipponbare reference genome. The number of total annotated SNPs was higher than the total number of quality SNPs due to more than one annotation in a single SNP.

	Bali	PH9	MRM16	MRQ100	Total
Number of raw SNPs	2,394,592	2,227,819	2,740,764	2,380,079	9,743,254
Number of high-quality SNPs	436,322	412,791	469,782	435,382	1,754,277
Intergenic SNPs	328,261	310,712	349,786	327,021	1,315,780
Genic SNPs	149,232	140,677	165,124	149,903	604,936

**Table 3:** Biological process and molecular function GO terms associated with genes containing SNPs with polymorphic sites. False discovery rate (FDR < 0.05). Only the top 10 GO terms from biological process and molecular function were further discussed in this paper.

Reactome pathway name	Molecular function GO terms	Frequency of genes containing SNPs with polymorphic sites	Biological process GO terms	Frequency of genes containing SNPs with polymorphic sites
(1) Metabolism and regulation (R-OSA-2744345) (2) Secondary metabolite biosynthesis (R-OSA-2744341) (3) Hormone biosynthesis, signalling, and transport (R-OSA-2744341)	Binding (GO:0005488)	55	Cellular process (GO:0009987)	51
	Catalytic activity (GO:0003824)	52	Metabolic process (GO:0008152)	49
	Heterocyclic compound binding (GO:1901363)	43	Organic substance metabolic process (GO:0071704)	44
	Organic cyclic compound binding (GO:0097159)	43	Primary metabolic process (GO:0044238)	41
	Ion binding (GO:0043167)	38	Cellular metabolic process (GO:0044237)	41
	Small molecule binding (GO:0036094)	24	Nitrogen compound metabolic process (GO:0006807)	37
	Nucleotide binding (GO:0000166)	24	Macromolecule metabolic process (GO:0043170)	34
	Nucleoside phosphate binding (GO:1901265)	24	Cellular macromolecule metabolic process (GO:0044260)	29
	Purine nucleotide binding (GO:0017076)	23	Macromolecule modification (GO:0043412)	20
	Carbohydrate derivative binding (GO:0097367)	23	Cellular protein modification process (GO:0006464)	18

**Table 4:** Annotation of nonsynonymous SNPs with polymorhic sites in Pfam family.

Functional gene classifications	Pfam name and ID	Number of SNPs
Stress responsive	AIG1 family (PF04548) Ubiquitin-conjugating enzyme (PF00179) NB-ARC domain (PF00931) Protein tyrosine kinase (PF07714)	5
Metabolism	Glycosyltransferase family 43 Cytochrome P450	2
Transporter	Mitochondrial carrier protein ABC transporter	2
Transcription factor	Myb-like DNA-binding domain AP2 domain IQ calmodulin-binding motif SNF2 family N-terminal domain	4

**Table 5:** Overview of genic SNPs in the genes encoding enzyme of flavonoid biosynthetic pathway. All genes were categorized into general phenylpropanoid, early biosynthetic genes, late biosynthetic genes, and transcription factor (bHLH (Kala4 and Rc), R2R3-MYB).

Group of genes	Genes name	Total SNPs	Total SNPs (%)
General phenylpropanoid genes	Phenylalanine ammonia-lyase (PAL) Cinnamate-4-hydroxylase (C4H) 4-Coumarate ligase (4CL)	511	28
Early biosynthetic genes (EBGs)	Chalcone synthase (CHS) Chalcone isomerase (CHI) Flavanone 3-hyroxylase (F3H) Flavanone 3′-hydroxylase (F3′H)	463	26
Late biosynthetic genes (LBGs)	Dihydroflavonol reductase (DFR) Leucoanthocyanidin reductase (LAR) UDP-glucose flavonoid 3-O-glucosyl transferase (UGT) Leucoanthocyanidin oxidase (LDOX)	675	37
Transcription factors (TFs)	Basic helix-loop-helix (bHLH) R2R3-MYB	160	9

## Data Availability

The raw sequencing reads data used to support the findings of this study have been deposited in the ENA database (https://www.ebi.ac.uk/ena). Accession numbers are ERR2831548(Bali), ERR2831549(PH9), ERR2831551(MRM16) and ERR2831550(MRQ100).

## Supplementary Materials

Supplementary Materials Supplementary Table 1: list of 99 flavonoid biosynthetic genes. Supplementary Table 2: list of nonsynonymous SNPs of 16 flavonoid biosynthesis genes in four pigmented rice varieties (Bali, PH9, MRM16, and MRQ100). Supplementary Figure 1: unique SNP shows the allele present in one variety whilst SNPs with polymorphic sites show the presence of SNP in each variety but with several allele combinations.
