# Supplementary material for: Discovery of Functional SNPs via Genome-Wide Exploration of Malaysian Pigmented Rice Varieties
Source: Int J Genomics. 2019 Oct 10;2019:4168045. doi: 10.1155/2019/4168045 (PMC6811786; doi:10.1155/2019/4168045)
Supplement: Supplementary Materials — Supplementary Table 1: list of 99 flavonoid biosynthetic genes. Supplementary Table 2: list of nonsynonymous SNPs of 16 flavonoid biosynthesis genes in four pigmented rice varieties (Bali, PH9, MRM16, and MRQ100). Supplementary Figure 1: unique SNP shows the allele present in one variety whilst SNPs with polymorphic sites show the presence of SNP in each variety but with several allele combinations. [file 4168045.f1.docx]

# Supplementary Table 1: List of 99 flavonoid biosynthetic genes and transcription factors that were selected from Nipponbare reference genome using similarity and bibliomic search. The flavonoid biosynthetic genes were categorized into three groups; i.e. i) general phenylpropanoid, ii) early biosynthetic genes and iii) late biosynthetic genes.

| **RAPDB OsID** | **UniProt ID** | **Flavonoid Biosynthetic Genes** | **Gene descriptions** | **Sources** |
| --- | --- | --- | --- | --- |
| ***General phenylpropanoid***  ***PAL*** |  |  |  |  |
| Os02g0626100 | P14717 | PAL | Similar to Phenylalanine ammonia-lyase. | 73 |
| Os02g0626400 | Q6K6Q1 | PAL | Phenylalanine ammonia-lyase (EC 4.3.1.24) | RiceCyc, RAPDB, OryzaCyc |
| Os02g0626600 | A0A0P0VM80 | PAL | Similar to Phenylalanine ammonia-lyase. | 7 |
| Os02g0627100 | Q0DZE0 | PAL | Similar to Phenylalanine ammonia-lyase (EC 4.3.1.5). | 73 |
| Os04g0518100 | Q7X720 | PAL | Phenylalanine ammonia-lyase. | RiceCyc, RAPDB, OryzaCyc |
| Os04g0518400 | Q7X8V3 | PAL | Similar to Phenylalanine ammonia-lyase (Fragment). | RiceCyc, RAPDB, OryzaCyc |
| Os05g0427400 | Q75HQ7 | PAL | Similar to Phenylalanine ammonia-lyase. | 73 |
| Os12g0520200 | Q0IMZ6 | PAL | Similar to phenylalanine ammonia-lyase. | RiceCyc, RAPDB, OryzaCyc |
| ***C4H*** |  |  |  |  |
| Os01g0820000 | A0A0P0V9R1 | C4H | Similar to Cinnamate-4-hydroxylase. | 73 |
| Os02g0467600 | Q6K7B1 | C4H | Similar to Cinnamate 4-hydroxylase CYP73. | RiceCyc, RAPDB, OryzaCyc |
| Os05g0320700 | Q5W6F1 | C4H | Similar to Cytochrome P450. | RiceCyc, RAPDB, OryzaCyc |
| Os06g0102100 | Q5VRI5 | C4H | Cytochrome P450 93G2 protein (CYP93G2), flavanone 2-hydroxylase (F2H), C-glycosylflavone biosynthesis | 68 |
| ***4CL*** |  |  |  |  |
| Os01g0901500 | A0A0P0VBX1 | 4CL | Similar to 4-coumarate--CoA ligase-like 5. | RiceCyc, RAPDB, OryzaCyc |
| Os01g0901600 | Q8RU95 | 4CL | Similar to 4-coumarate--CoA ligase-like 6. | RiceCyc, RAPDB, OryzaCyc |
| Os02g0177600 | Q6ETN3 | 4CL | 4-coumarate:coenzyme A ligase, Lignin biosynthesis, Defense against wounding | RiceCyc, RAPDB, OryzaCyc |
| Os02g0697400 | Q42982 | 4CL | 4-coumarate:coenzyme A ligase, Flavonoid biosynthesis | 73 |
| Os03g0132000 | Q10S72 | 4CL | Similar to 4-coumarate-CoA ligase-like protein. | RiceCyc, RAPDB, OryzaCyc |
| Os03g0152400 | Q0DV32 | 4CL | Similar to 4-coumarate--CoA ligase-like 1. | RiceCyc, RAPDB, OryzaCyc |
| Os06g0656500 | Q67W82 | 4CL | 4-coumarate:coenzyme A ligase, Lignin biosynthesis, Defense against wounding | RiceCyc, RAPDB, OryzaCyc |
| Os07g0280200 | Q69RG7 | 4CL | AMP-dependent synthetase and ligase domain containing protein. | RiceCyc, RAPDB, OryzaCyc |
| Os08g0143300 | Q6YYZ2 | 4CL | AMP-dependent synthetase and ligase domain containing protein. | RiceCyc, RAPDB, OryzaCyc |
| Os08g0245200 | P17814 | 4CL | 4-coumarate:coenzyme A ligase, Lignin biosynthesis | 73 |
| Os08g0448000 | Q6ZAC1 | 4CL | 4-coumarate:coenzyme A ligase, Lignin biosynthesis, Defense against wounding | RiceCyc, RAPDB, OryzaCyc |
| Os10g0578950 | A0A0P0XXS5 | 4CL | Similar to 4-coumarate--CoA ligase-like 2. | RiceCyc, RAPDB, OryzaCyc |
| **Early biosynthetic genes**  ***CHS*** |  |  |  |  |
| Os07g0214900 | Q8H4L3 | CHS | Similar to Chalcone synthase C2 (EC 2.3.1.74) (Naringenin-chalcone synthase C2). | 74 |
| Os11g0530600 | Q2R3A1 | CHS | Similar to Chalcone synthase C2 (EC 2.3.1.74) (Naringenin-chalcone synthase C2). | 75 |
| Os04g0103900 | Q7XMR1 | CHS | Similar to Chalcone synthase. | RiceCyc, RAPDB, OryzaCyc |
| Os05g0212900 | Q0DJY7 | CHS | Similar to Chalcone synthase J (EC 2.3.1.74) (Naringenin-chalcone synthase J). | RiceCyc, RAPDB, OryzaCyc |
| Os07g0271500 | Q8LIL0 | CHS | Similar to Chalcone synthase. | RiceCyc, RAPDB, OryzaCyc |
| Os07g0501100 | Q6ZIJ3 | CHS | Similar to Chalcone synthase 2 (EC 2.3.1.74) (Naringenin-chalcone synthase 2). | RiceCyc, RAPDB, OryzaCyc |
| Os07g0525500 | Q69UB7 | CHS | Similar to chalcone synthase 8. | RiceCyc, RAPDB, OryzaCyc |
| ***CHI*** |  |  |  |  |
| Os03g0819600 | Q84T92 | CHI | Chalcone isomerase (EC 5.5.1.6). | 73,76 |
| Os02g0320300 | Q6EQW2 | CHI | Similar to Chalcone isomerase 3. | RiceCyc, RAPDB, OryzaCyc |
| Os02g0778500 | Q6K7H0 | CHI | Chalcone isomerase domain containing protein. | RiceCyc, RAPDB, OryzaCyc |
| Os06g0203600 | Q69SP9 | CHI | Chalcone isomerase domain containing protein. | RiceCyc, RAPDB, OryzaCyc |
| Os07g0571600 | Q0D5A7 | CHI | Chalcone isomerase domain containing protein. | RiceCyc, RAPDB, OryzaCyc |
| Os11g0116300 | Q2RBC7 | CHI | Chalcone isomerase domain containing protein. | RiceCyc, RAPDB, OryzaCyc |
| Os12g0115700 | Q2QYK6 | CHI | Chalcone isomerase domain containing protein. | RiceCyc, RAPDB, OryzaCyc |
| ***F3H*** |  |  |  |  |
| Os04g0662600 | Q7XM21 | F3H | Similar to Naringenin,2-oxoglutarate 3-dioxygenase. | 54 |
| Os10g0320100 | Q7G602 | F3'H | Similar to Flavonoid 3'-monooxygenase (EC 1.14.13.21) (Flavonoid 3'-hydroxylase) (Cytochrome P450 75B2). | 74 |
| Os10g0536400 | Q8W2X5 | F3H | Similar to Oxidoreductase, 2OG-Fe oxygenase family protein, expressed. | 54 |
| Os03g0122300 | Q8H8H6 | F3H | Similar to Flavanone 3-hydroxylase-like protein. | RiceCyc, RAPDB, OryzaCyc |
| Os04g0581000 | B9FC47 | F3H | Similar to Flavanone 3-hydroxylase (Fragment). | RiceCyc, RAPDB, OryzaCyc |
| Os10g0317900 | Q8LM92 | F3H | Chrysoeriol 5'-Hydroxylase, Flavonoid B-ring hydroxylase, Tricin biosynthesis | 73 |
| Os10g0559500 | Q7XC75 | F3H | 2OG-Fe(II) oxygenase domain containing protein. | RiceCyc, RAPDB, OryzaCyc |
| **F3'H** |  |  |  |  |
| Os10g0320100 | Q7G602 | F3'H | Similar to Flavonoid 3'-monooxygenase (EC 1.14.13.21) (Flavonoid 3'-hydroxylase) (Cytochrome P450 75B2). | 74 |
| ***Late biosynthetic genes***  ***DFR*** |  |  |  |  |
| Os01g0633500 | Q9S7C3 | DFR | Similar to Dihydroflavonol reductase. | 65 |
| Os03g0184600 | A0A0P0VU49 | DFR | Similar to Dihydroflavonol-4-reductase. | RiceCyc, RAPDB, OryzaCyc |
| Os09g0491868 | A0A0P0XNV1 | DFR | Similar to dihydroflavonol-4-reductase. | 16 |
| Os03g0184550 | Q94HG6 | DFR | Similar to Dihydroflavonol-4-reductase. | RiceCyc, RAPDB, OryzaCyc |
| Os08g0183900 | Q0J7K9 | DFR | Similar to dihydroflavonol-4-reductase. | RiceCyc, RAPDB, OryzaCyc |
| Os08g0515900 | A0A0N7KQ50 | DFR | Similar to Dihydroflavonol-4-reductase. | RiceCyc, RAPDB, OryzaCyc |
| Os09g0491820 | C7J743 | DFR | Similar to dihydroflavonol-4-reductase. | RiceCyc, RAPDB, OryzaCyc |
| Os09g0491820 | A0A0N7KR15 | DFR | Similar to dihydroflavonol-4-reductase. | RiceCyc, RAPDB, OryzaCyc |
| ***LAR*** |  |  |  |  |
| Os03g0259400 | Q3KN74 | LAR | Similar to Leucoanthocyanidin reductase (EC 1.17.1.3) (Leucocyanidin reductase). | RiceCyc, RAPDB, OryzaCyc |
| Os03g0289800 | Q10MY3 | LAR | Similar to Leucoanthocyanidin dioxygenase-like protein. | RiceCyc, RAPDB, OryzaCyc |
| Os04g0630800 | Q0J9U6 | LAR | Similar to Anthocyanidin reductase. | RiceCyc, RAPDB, OryzaCyc |
| ***LDOX*** |  |  |  |  |
| Os01g0372500 | Q93VC3 | LDOX | Similar to Leucoanthocyanidin dioxygenase (EC 1.14.11.19) (LDOX) (Leucocyanidin oxygenase) (Leucoanthocyanidin hydroxylase). | 73,74 |
| Os06g0626700 | Q67VR7 | LDOX | Isopenicillin N synthase family protein. | 73,74 |
| Os06g0176850 | A0A0P0WTN5 | LDOX | Similar to leucoanthocyanidin dioxygenase. | RiceCyc, RAPDB, OryzaCyc |
| Os06g0178650 | A3B8Y2 | LDOX | Similar to leucoanthocyanidin dioxygenase. | RiceCyc, RAPDB, OryzaCyc |
| Os01g0832600 | Q5QLC8 | LDOX | Similar to Leucoanthocyanidin dioxygenase-like protein. | RiceCyc, RAPDB, OryzaCyc |
| Os05g0127500 | Q75IL5 | LDOX | Similar to Leucoanthocyanidin dioxygenase-like protein. | RiceCyc, RAPDB, OryzaCyc |
| Os06g0177700 | Q0DE49 | LDOX | Similar to leucoanthocyanidin dioxygenase. | RiceCyc, RAPDB, OryzaCyc |
| Os07g0148200 | Q6ZF65 | LDOX | Similar to Flavonol 3-O-glucosyltransferase (EC 2.4.1.91) (UDP-glucose flavonoid 3-O-glucosyltransferase) (Bronze-1) (Bz-McC allele). | RiceCyc, RAPDB, OryzaCyc |
| Os09g0353400 | A0A0P0XKP2 | LDOX | Similar to Leucoanthocyanidin dioxygenase. | RiceCyc, RAPDB, OryzaCyc |
| Os09g0353700 | A0A0N7KQM8 | LDOX | Similar to Leucoanthocyanidin dioxygenase. | RiceCyc, RAPDB, OryzaCyc |
| Os09g0354100 | A0A0P0XL25 | LDOX | Similar to Leucoanthocyanidin dioxygenase. | RiceCyc, RAPDB, OryzaCyc |
| Os06g0178450 | A0A0P0WTI2 | LDOX | Similar to Leucoanthocyanidin dioxygenase. | RiceCyc, RAPDB, OryzaCyc |
| Os06g0176500 | A0A0P0WTN5 | LDOX | Similar to leucoanthocyanidin dioxygenase. | RiceCyc, RAPDB, OryzaCyc |
| Os06g0176650 | A0A0P0WTI2 | LDOX | Similar to leucoanthocyanidin dioxygenase. | RiceCyc, RAPDB, OryzaCyc |
| ***UGT*** |  |  |  |  |
| Os01g0736100 | Q942B6 | UGT | UDP-glucuronosyl/UDP-glucosyltransferase family protein. | 68 |
| Os01g0736300 | Q942B3 | UGT | Similar to anthocyanidin 5,3-O-glucosyltransferase. | 68 |
| Os02g0483500 | Q6K2J2 | UGT | Transferase family protein. | 68 |
| Os02g0589400 | Q0DZZ5 | UGT | UDP-glucuronosyl/UDP-glucosyltransferase family protein. | 77 |
| Os05g0527000 | Q65X84 | UGT | UDP-glucuronosyl/UDP-glucosyltransferase family protein. | 68 |
| Os06g0192100 | Q69TJ1 | UGT | Similar to UDP-glucose flavonoid-3-O-glucosyltransferase. | RiceCyc, RAPDB, OryzaCyc |
| Os06g0288300 | Q5VMI0 | UGT | C-glucosyltransferase, Flavone-C-glycoside synthesis | 68 |
| Os07g0243000 | A0A0P0X4K8 | UGT | Similar to UDP-glycosyltransferase UGT710F3. | RiceCyc, RAPDB, OryzaCyc |
| Os01g0176000 | Q9FU69 | UGT | UDP-glucuronosyl/UDP-glucosyltransferase family protein. | RiceCyc, RAPDB, OryzaCyc |
| Os01g0597800 | Q5ZAF2 | UGT | UDP-glucuronosyl/UDP-glucosyltransferase family protein. | RiceCyc, RAPDB, OryzaCyc |
| Os01g0598000 | A0A0P0V4U0 | UGT | Similar to UDP-glycosyltransferase UGT703A5. | RiceCyc, RAPDB, OryzaCyc |
| Os01g0638000 | Q94CZ1 | UGT | UDP-glucuronosyl/UDP-glucosyltransferase family protein. | RiceCyc, RAPDB, OryzaCyc |
| Os01g0734600 | Q942C5 | UGT | UDP-glucuronosyl/UDP-glucosyltransferase family protein. | RiceCyc, RAPDB, OryzaCyc |
| Os01g0735300 | Q0JJJ6 | UGT | UDP-glucuronosyl/UDP-glucosyltransferase family protein. | RiceCyc, RAPDB, OryzaCyc |
| Os04g0206500 | Q7XWK3 | UGT | UDP-glucuronosyl/UDP-glucosyltransferase family protein. | RiceCyc, RAPDB, OryzaCyc |
| Os05g0215300 | Q6I5X0 | UGT | UDP-glucuronosyl/UDP-glucosyltransferase family protein. | RiceCyc, RAPDB, OryzaCyc |
| Os05g0499800 | Q6AUW6 | UGT | UDP-glucuronosyl/UDP-glucosyltransferase family protein. | RiceCyc, RAPDB, OryzaCyc |
| Os05g0500000 | Q0DGZ9 | UGT | UDP-glucuronosyl/UDP-glucosyltransferase family protein. | RiceCyc, RAPDB, OryzaCyc |
| Os05g0527600 | A0A0P0WQ19 | UGT | Similar to anthocyanidin 5,3-O-glucosyltransferase. | RiceCyc, RAPDB, OryzaCyc |
| Os05g0527800 | Q65XD0 | UGT | UDP-glucuronosyl/UDP-glucosyltransferase family protein. | RiceCyc, RAPDB, OryzaCyc |
| Os06g0343600 | Q5Z9X3 | UGT | UDP-glucuronosyl/UDP-glucosyltransferase family protein. | RiceCyc, RAPDB, OryzaCyc |
| Os07g0241800 | Q7XI34 | UGT | UDP-glucuronosyl/UDP-glucosyltransferase family protein. | RiceCyc, RAPDB, OryzaCyc |
| Os11g0444000 | Q53KZ0 | UGT | Similar to UDP-glucosyltransferase BX8. | RiceCyc, RAPDB, OryzaCyc |
| Os11g0599200 | Q2R1N0 | UGT | UDP-glucuronosyl/UDP-glucosyltransferase family protein. | RiceCyc, RAPDB, OryzaCyc |
| **Regulatory genes** |  |  |  |  |
| Os04g0557500 | A0A0P0WDK6 | Kala4 | Similar to Transcriptional activator Ra homolog (Fragment). | 63,78 |
| Os06g0205100 | E9MZX3 | R2-R3 MYB | Similar to Transcriptional activator. | 79 |
| Os04g0557800 | Q7XPS3 | OsB1 | Similar to R-type basic helix-loop-helix protein. | 78 |
| Os07g0211500 | A0A0P0X425 | Rc | Similar to BHLH protein. | 64 |

Supplementary Table 2. List of 40 non-synonymous SNPs in 39 flavonoid biosynthetic genes and one transcription factor. Table 2 describes SNPs information (i.e. SNP identifier (SNP ID), gene identifier, reference allele, SNP allele for each varieties (Bali, PH9, MRM16 and MRQ100), chromosome and SNP position.

| **Genes** | **RAPDB ID** | **Chromosome** | **SNPs Position** | **Reference Allele**  **Nipponbarre** | **SNP ID** | **Bali** | **PH9** | **MRM16** | **MRQ100** |
| --- | --- | --- | --- | --- | --- | --- | --- | --- | --- |
| Cinnamate-4-hydroxylase (C4H) | Os06g0102100 | chr06 | 163782 | A | m_C4H_1 | G | - | - | - |
| 4-coumarate ligase (4CL) | Os01g0901600  Os02g0697400  Os02g0177600  Os06g0656500  Os08g0245200  Os10g0578950  Os07g0280200 | chr01  chr02  chr02  chr06  chr08  chr10  chr07 | 39258803  28680568  4282355  26943434  8878252  23085559  10637458 | C  G  A  A  G  G  C | b_4CL_1  b_4CL_2  m_4CL_3  m_4CL_4  m_4CL_5  m_4CL_6  m_4CL_7 | T  A  -  -  -  -  - | -  -  -  -  -  -  - | -  -  G  G  A  T  - | -  -  -  -  -  -  G |
| Chalcone synthase (CHS) | Os11g0530600  Os04g0103900  Os07g0271500  Os07g0525500 | chr11  chr04  chr07  chr07 | 19280813  259218  10018833  20421061 | C  C  G  C | b_CHS_1  b_CHS_2  m_CHS_3  m_CHS_4 | T  -  -  - | -  G  -  - | -  -  -  - | -  -  A  T |
| Chalcone isomerase (CHI) | Os02g0778500  Os03g0819600  Os02g0320300 | chr02  chr03  chr02 | 32958590  34395506  12771657 | G  C  G | b_CHI_1  b_CHI_2  b_CHI_3 | A  T  - | -  -  T | -  -  - | -  -  - |
| Dihydroflavonol reductase (DFR) | Os08g0183900  Os08g0183900  Os09g0491820 | chr08  chr08  chr09 | 4900215  4900194  19012056 | G  G  A | b_DFR_1  m_DFR_2  m_DFR_3 | A  -  - | -  -  - | -  C  G | -  -  - |
| UDP-glycosyl transferase (UGT) | Os05g0527000  Os06g0288300  Os01g0597800  Os01g0598000  Os04g0206500  Os05g0527000  Os01g0176000  Os05g0527800  Os07g0243000  Os11g0444000  Os01g0598000  Os05g0527000  Os05g0527000 | chr05  chr06  chr01  chr01  chr04  chr05  chr01  chr05  chr07  chr11  chr01  chr05  chr05 | 26199529  10479849  23456938  23465058  7155912  26199448  3920003  26227130  7969514  14673251  23465067  26199225  26199416 | A  A  T  A  G  C  T  T  C  G  T  C  A | b_UGT_1  b_UGT_2  b_UGT_3  b_UGT_4  b_UGT_5  b_UGT_6  m_UGT_7  m_UGT_8  m_UGT_9  m_UGT_10  m_UGT_11  m_UGT_12  m_UGT_13 | G  G  -  -  -  -  -  -  -  -  -  -  - | -  -  G  T  T  T  -  -  -  -  -  -  - | -  -  -  -  -  -  C  C  G  T  -  -  - | -  -  -  -  -  -  -  -  -  -  G  T  C |
| Leucoanthocyanidin dioxygenase (LDOX) | Os05g0127500  Os07g0148200  Os09g0353400  Os09g0354100  Os05g0527600  Os07g0148200  Os09g0354100  Os07g0148200 | chr05  chr07  chr09  chr09  chr05  chr07  chr09  chr07 | 1566532  2490069  11288999  11322916  26218022  2490225  11322892  2489295 | C  G  T  C  T  G  C  A | b_LDOX_1  b_LDOX_2  b_LDOX_3  b_LDOX_4  m_LDOX_1  m_LDOX_2  m_LDOX_3  m_LDOX_4 | G  A  G  T  -  -  -  - | -  -  -  -  -  -  -  - | -  -  -  -  C  T  A  - | -  -  -  -  -  -  -  C |
| R2R3-MYB (TF) | Os01g0305900 | chr01 | 11356196 | G | b_MYB_1 | - | A | - | - |


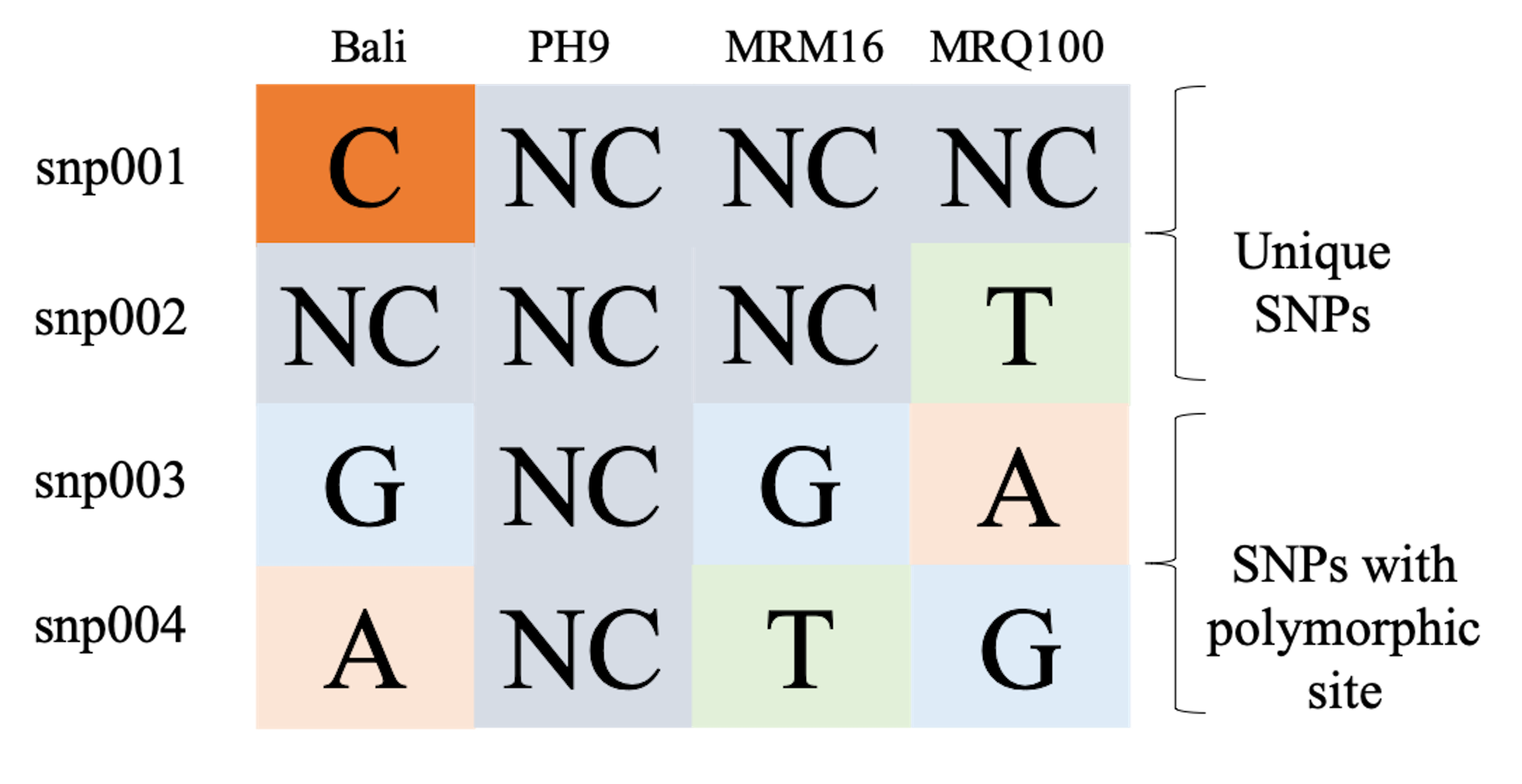


Supplementary Figure 1. Unique SNPs show the allele presence in one variety whilst SNPs with polymorphic sites show the presence of SNP in each variety but with several alleles combination.
